# Constructing a Sr^2+^-Substituted Surface Hydroxyapatite Hexagon-Like Microarray on 3D-Plotted Hydroxyapatite Scaffold to Regulate Osteogenic Differentiation

**DOI:** 10.3390/nano10091672

**Published:** 2020-08-26

**Authors:** Yingqi Wei, Huichang Gao, Lijing Hao, Xuetao Shi, Yingjun Wang

**Affiliations:** 1 Department of Biomedical Engineering, School of Materials Science and Engineering, South China University of Technology, Guangzhou 510641, China; wyq0215@foxmail.com; 2 National Engineering Research Center for Tissue Restoration and Reconstruction, Guangzhou 510006, China; haolijing709@163.com; 3 School of Medicine, South China University of Technology, Guangzhou 510006, China; mchcgao@scut.edu.cn; 4 Guangdong Province Key Laboratory of Biomedical Engineering, South China University of Technology, Guangzhou 510006, China

**Keywords:** 3D plotting, hydroxyapatite scaffold, hexagon-like microarray, Sr^2+^-substituted, osteogenic differentiation

## Abstract

Surface topography and chemical characteristics can regulate stem cell proliferation and differentiation, and decrease the bone-healing time. However, the synergetic function of the surface structure and chemical cues in bone-regeneration repair was rarely studied. Herein, a strontium ion (Sr^2+^)-substituted surface hydroxyapatite (HA) hexagon-like microarray was successfully constructed on 3D-plotted HA porous scaffold through hydrothermal reaction to generate topography and chemical dual cues. The crystal phase of the Sr^2+^-substituted surface microarray was HA, while the lattice constant of the Sr^2+^-substituted microarray increased with increasing Sr^2+^-substituted amount. Sr^2+^-substituted microarray could achieve the sustainable release of Sr^2+^, which could effectively promote osteogenic differentiation of human adipose-derived stem cells (ADSCs) even without osteogenic-induced media. Osteogenic characteristics were optimally enhanced using the higher Sr^2+^-substituted surface microarray (8Sr-HA). Sr^2+^-substituted microarray on the scaffold surface could future improve the osteogenic performance of HA porous scaffold. These results indicated that the Sr^2+^-substituted HA surface hexagon-like microarray on 3D-plotted HA scaffolds had promising biological performance for bone-regeneration repair scaffold.

## 1. Introduction

For large bone defects caused by severe fractures, tumor resection, or osteonecrosis, bone tissue itself cannot fully repair and regenerate [1,2]. Current clinical treatments of bone defects are associated with unavoidable immunogenicity issues, possible disease transmission, and other complications of the bone extraction area, which limit their wide application. Tissue engineering could effectively break through the barriers of existing treatment methods and improve bone tissue repair. During the repair of damaged bones, bone repair scaffold as carrier for repairing bone defects can provide effective mechanical support [3]. In addition, scaffold serves as a bridge between natural tissue and seed cells, providing spatial structure and growth templates for cell adhesion, proliferation and differentiation in bone regeneration and repair with the potential to regulate cell activity and fate [4,5]. Therefore, material selection and structural design of scaffold are key considerations in the process of bone repair.

Recent studies have mainly focused on metals, polymers, bioglass, bioceramic and their hybrid materials as scaffold materials [6]. Meanwhile, bioceramic materials are similarly to the inorganic component of natural bone tissue, and have good biocompatibility and bioactivity [7,8], making materials such as hydroxyapatite (HA) and tricalcium phosphate (TCP) the most promising bone repair materials [9]. In addition, the degradation products of calcium and phosphorus materials and the released ions can participate in normal metabolic activities of the human body, enhance cell activity and accelerate the bone repair process [10,11,12].

In addition to the choice of scaffold material for designing a perfect bone repair scaffold, the surface characteristics (physical structure and chemical properties) are also of particularly interest to researchers [13,14]. Among them, surface morphology and chemical composition are critical parameters that should be considered. However, few studies have considered the synergetic functions of scaffold surface morphology and chemical characteristics guiding biological responses. It has been reported that cells can directly sense the structural characteristics of the scaffold surface and respond [15], which can further regulate the fate of stem cells and their differentiation into different cell lineages in tissue engineering repair [16].

For the application of bioceramic in bone tissue engineering, most studies have focused on controlling the crystal size [17], pore size [18,19], pore structure [5,20], porosity [21,22], and surface topography [23,24]. It was confirmed that bioceramic scaffolds with pore sizes > 200 µm are necessary to enhance bone formation [13]. HA bioceramic disks with hierarchical micro/nanohybrid surface topographies can result in better outcomes by simultaneously facilitating of protein adsorption, cell proliferation and osteogenic differentiation [24]. Micro/nanohybrid surface topographies can also accelerate the new bone formation and mineralization of HA bioceramic, showing enormous potential for their ability to improve the clinical performance of macroporous HA bioceramic [23]. However, as bioceramic has low processability and high brittleness, it is different to fabricate uniform topographic structures on 3D porous scaffold surfaces. Meanwhile, human adipose-derived stem cells (ADSCs) is easier to harvest and showed a better proliferation and osteogenic differentiation capabilities compared to Bone Mesenchymal Stem Cells (BMSCs) [25]. In addition, ADSCs displayed no significant deficiencies in immunomodulatory potential [26]. Our previous research built a uniform HA hexagon-like microarray on the whole surface of 3D-plotted HA scaffold. In addition, the results showed that the microarray structure had a good cytocompatibility with human ADSCs and promoted the expression levels of osteogenic-related genes and proteins of ADSCs [27].

Surface structure of scaffold can regulate the proliferation and differentiation of stem cells; similarly, chemical cues from bioceramic scaffold also exert guiding effects on the osteogenic differentiation of stem cells [28,29]. Strontium, silicon, magnesium, zinc, etc., are trace elements in human bones, and their doping into bioceramic scaffold as functional inorganic ions can promote the transformation of cell functions and lead to bone and vascular regeneration [11,30]. It has been revealed that Sr^2+^ stimulates bone formation through a dual mode of action, enhances the osteogenic activity of osteoblasts, and inhibits bone resorption by osteoclasts [31]. In the preparation of bone tissue engineering scaffolds, the introduction of Sr^2+^ can promote bone regeneration [32]. However, whether the HA microarray and the Sr^2+^ released from the microarray have a synergistic function in the osteogenic differentiation of stem cells needs to be further investigated.

The primary aim of this study was to evaluate the effect of Sr^2+^-substituted microarray on the physicochemical properties and osteogenic capacity of 3D-plotted HA porous scaffold. On the basic of a previous study [27], surface HA hexagon-like microarray substituted with varying amounts of Sr(NO_3_)_2_ (0–8 mol% Sr^2+^) was performed on HA scaffold by hydrothermal reaction. The surface topography, phase composition, and Sr^2+^ releasing rate of the microarray were evaluated in vitro. The ability of Sr ions released from the microarray to modulate the proliferation and differentiation of ADSCs was also investigated.

## 2. Materials and Methods

### 2.1. Construction of HA Scaffolds

**Figure 1:**
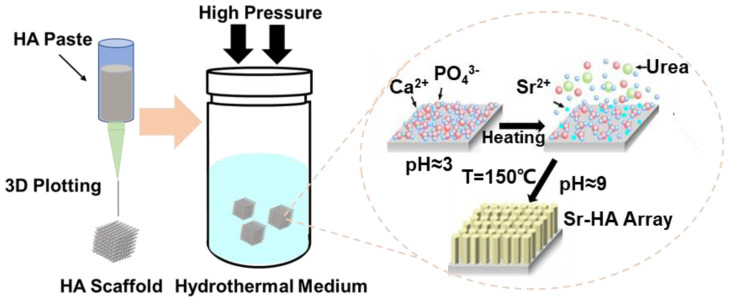
Schematic illustration of the topography of the HA scaffolds and their effects on osteogenic differentiation.

HA powder and 3D plotted HA scaffolds were fabricated in sequence, according the previous experimental procedure [24]. The Sr^2+^-substituted hexagon-like microarray was constructed on 3D-plotted HA scaffolds via hydrothermal synthesis reaction. First, the Sr^2+^-substituted amount was controlled at 0%, 1%, 4% and 8% *M_Sr/(Ca+Sr)_* (molar ratio of Sr(NO_3_)_2_ (Guangzhou Chemical Co., Guangzhou, China) compared to Ca(NO_3_)_2_ (Guangzhou Chemical Co., Guangzhou, China) and Sr(NO_3_)_2_, *M_Sr/(Ca+Sr)_* = *M_Sr_*/(*M_Sr_* + *M_Ca_*) · 100%) in the progress of hydrothermal reaction. In the hydrothermal medium, the total concentrations of Ca(NO_3_)_2_·4H_2_O and Sr(NO_3_)_2_ were kept at 0.3 M, the concentration of (NH_4_)_2_HPO_4_ (Guangzhou Chemical Co., Guangzhou, China) was kept at 0.18 M fix the *M_(Ca+Sr)/P_* at 1.67. The medium pH was maintained at 2.6 ~ 3.0 with dilute nitric acid to keep the medium clear. Then, urea (3 g) (CO(NH_2_)_2_, Guangzhou Chemical Co., Guangzhou, China) was dissolved into the medium (50 mL). Next, the 3D-plotted HA scaffolds and the medium were poured into an autoclave and heated to 150 °C for 3 h. During this process, the urea was decomposed to keep the medium pH at 8.0 ~ 9.0, which was necessary for the construction of the hexagon-like microarray on the HA scaffold surface (Figure 1). The prepared Sr^2+^-substituted HA hexagon-like microarray based on 3D plotting hydroxyapatite scaffolds were named 0Sr-HA, 1Sr-HA, 4Sr-HA, and 8Sr-HA.

### 2.2. Characterization of HA Scaffolds

The morphology and surface structure of the Sr^2+^-substituted HA scaffolds were analyzed by field emission scanning electron microscopy (FE-SEM, Merlin, Goettingen, Germany). The element distribution on HA scaffold surface was tested by X-ray energy dispersive spectrometer (EDS, AMETEK, PA, USA). The scaffolds phase was detected by X-ray diffraction (XRD, Empyrean, Almelo, The Netherlands) and was further analyzed by X’Pert HighScore and MDI Jade 6 software. The scaffolds were immersed in cell culture medium, and the cumulative concentration of ions released into the medium was evaluated by Inductively Coupled Plasma (ICP, Optimal 5300DV, MA, USA).

### 2.3. Cell Culture

ADSCs (Cyagen, CA, USA) were used to assess the cytocompatibility of the Sr^2+^-substituted HA scaffolds. ADSCs were cultured in 37 °C standing-temperature cultivator with 5% CO_2_, and cells at early passages (≤6) were used throughout the study. When ADSCs were spread over 80% of the culture flask, the cells were passaged or transplanted onto the surface of the scaffold. The cell medium was replaced every other day for the duration of the experiment.

### 2.4. Cell Cytocompatibility

Cell cytocompatibility of the Sr^2+^-substituted HA microarray on the 3D-plotted HA scaffolds was evaluated by CCK-8 assay kit and Live/dead staining according to the manufacturer’s instructions. Briefly, autoclaved scaffolds were soaked in ADSC culture medium for 8 h. ADSCs (1 × 10^5^ cells/scaffold) were implanted on the preprocessed scaffolds and cultivated for 7 days. At specific culture times (1, 4, and 7 days), the cells on the scaffolds were subjected to CCK-8 assay, and their absorbance at 450 nm of them was evaluated via a microplate reader (Thermo 3001, MA, USA). The cells were also stained with Live-dead kit and analyzed by confocal laser scanning microscopy (CLSM, Leica SP8, Weztlar, Germany). The cytoskeleton fixed on Sr^2+^-substituted scaffold surface was stained and detected via CLSM. ADSCs on the HA scaffold were fixed, gradient dehydrated by ethyl alcohol (30%, 50%, 75%, 95%, 100%, 100%, 15 min/time) and lyophilized. Then cell spreading on the HA scaffolds was evaluated by FE-SEM.

### 2.5. Cell Differentiation Behavior

The osteogenic differentiation behavior of ADSCs on Sr^2+^-substituted HA scaffolds was evaluated via qRT-PCR analysis and immunofluorescent staining. In brief, pretreated Sr^2+^-substituted HA scaffolds were seeded into ADSCs (2 × 10^5^ cells/scaffold) and incubated in a humidified incubator for 14 days. The total Ribose Nucleic Acid (RNA)of the cells on the scaffolds was isolated, quantified, and reverse-transcribed into cDNA at specific incubation times (4, 7, and 14 days). Next, cDNA was amplified and normalized to calculate the expression levels of traditional osteogenic genes (ALP, BMP2, RUNX2, OCN, Osterix and VEGF) via QuantStudio^®^ 6 Flex instrument (Life Technologies, NY, USA). The qRT-PCR primer sequences were designed by TaKaRa Biotechnology (Osaka, Japan) and listed in Table S1. RUNX2 protein secreted by ADSCs was specifically labelled by immunofluorescent staining and detected via CLSM to further assess the effect of the Sr^2+^-substituted surface microarray on ADSCs differentiation behavior.

### 2.6. Statistical Analysis

The quantitative experimental results were expressed as mean ± standard deviation. Comparison between two means was evaluated by the t-test. A value of *p* < 0.05 considered to be statistically significant (* *p* < 0.05, ** *p* < 0.01, *** *p* < 0.001).

## 3. Results and Discussion

### 3.1. Characterization of the HA Scaffolds with Sr^2+^-Substituted Microarray

In the process of bone tissue repair, 3D interconnected macropore structures of the scaffolds are necessary for the delivery of nutrients and metabolic wastes, and is beneficial for cell growth and migration. We first prepared HA powder to develop a homogeneous paste with appropriate viscosity, and then fabricated the 3D-plotted HA porous scaffold with interconnected macropore structures [18,27]. As shown in Figure S1, HA scaffolds with various structures (such as shape, size, and pore structure) were fabricated by 3D plotting. The FE-SEM results (Figure S2) also proved that the HA scaffolds had interconnected pores and that the surface was clean and smooth. The HA grains were closely arranged, with a uniform size distribution. The XRD and EDS (Figure S3) results confirmed that the phase composition of the scaffold corresponded HA crystal.

**Figure 2:**
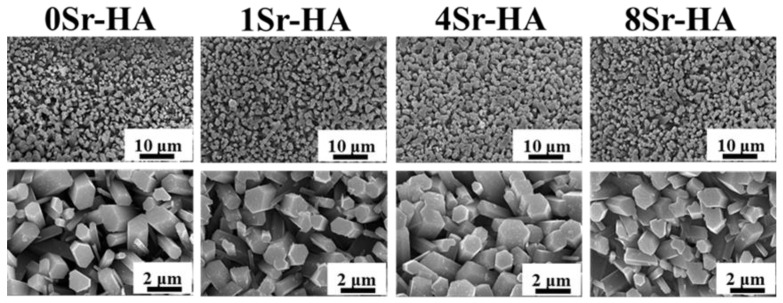
FE-SEM images of the HA hexagon-like microarray with different Sr^2+^-substituted content topographies on the HA scaffold surface.

Based on the fabrication of the 3D-plotted HA scaffold, Sr^2+^-substituted HA hexagon-like microarray was constructed by hydrothermal reaction, which is the recrystallization progress of Ca^2+^ and PO_4_^3-^ [33]. At the beginning of the hydrothermal reaction, the fresh smooth HA scaffolds were eroded in an acidic environment. Meanwhile, constituent ions of hydroxyapatite were released into the solution, which lead to the increase in the local Ca^2+^ and PO_4_^3−^ ions concentrations. As time went on, the solution became alkaline because of the decomposition of urea at high temperature. The Ca^2+^ and PO_4_^3−^ ions concentrations reached oversaturation, and HA crystals began to recrystallize on the eroded scaffold surfaces and acted as nucleation sites. The topographies of HA microarray on the scaffold with different Sr^2+^-substituted contents were showed in Figure 2. The results showed that the uniform hexagon-like microarray on the scaffold surface was no obvious change with increasing Sr^2+^ substituted amount. The fracture surface morphology of HA scaffolds (Figure S4) also showed that the microarray was successfully constructed on the surfaces of HA scaffolds and even on the internal surfaces of the scaffold; the length of the microarray was approximately 2–4 μm. The EDS results (Figure S5) of the Sr^2+^-substituted microarray on HA scaffold surfaces demonstrated that Sr^2+^ was evenly distributed on the scaffold surfaces and the *M_(Ca+Sr)/P_* value of the scaffold surfaces was close to the *M_Ca/P_* value of HA crystal (Ca_10_(PO_4_)_6_(OH)_2_).

**Figure 3:**
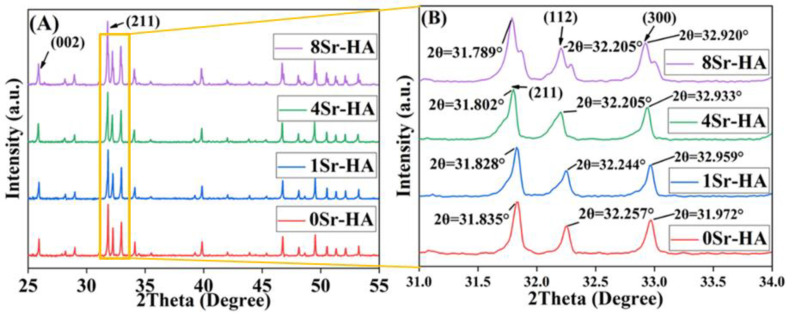
(**A**) XRD analysis of the HA hexagon-like microarray topography with different Sr^2+^-substituted content topographies on the HA scaffold surface. (**B**) is the enlarge figure about (**A**).

The crystalline structure of the scaffold surface microarray was detected by XRD test, and the results are shown in Figure 3. Compared with the HA standard PDF card (No.072-1243), the characteristic peak position (2θ) of the scaffold surface microarray corresponded to that of the standard card, and the spatial groups were P63/m (176) and hexagonal crystal system, indicating that the Sr^2+^-substitution had no significant influence on the phase composition of the surface microarray. As shown in Figure 3A, with increasing Sr^2+^-substituted amount in the surface microarray, the intensity of the diffraction peak was gradually increased. The amplification of the main characteristic peak in Figure 3A showed that the position of the characteristic peak moved to the left as the Sr^2+^-substituted amount increased, as shown in Figure 3B. In addition, the lattice constant changes of the surface microarray were further quantitatively analyzed by MDI Jade software, as shown in Table S3. With increasing Sr^2+^-substituted amount, the lattice constants (a and b directions) and lattice volume of the microarray crystal gradually increased. The ion radius of Sr^2+^ (113 pm) was higher than that of Ca^2+^ (99 pm), and Sr^2+^-substituted the Ca^2+^ position in the HA crystal, resulting an increase in the HA lattice constant [34,35]. The shift in the lattice constant indicated that during the formation of the Sr^2+^-substituted surface microarray, Sr^2+^ partially replaces the lattice position of Ca^2+^ in the HA crystals [34].

**Figure 4:**
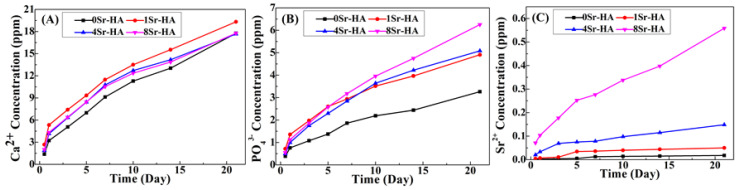
The cumulative concentration of ions ((**A**), Ca^2+^; (**B**), PO_4_^3-^; (**C**), Sr^2+^) released from the HA scaffolds with different Sr^2+^-substituted contents corresponding to different hexagon-like microarray topographies on the surface. Ions were released into the medium as a function of immersion time in cell culture medium.

To evaluate the Sr^2+^-substituted content on the HA scaffold surface, the *M_Sr/(Ca+Sr)_* value was further calculated and the results are shown in Table S2. The *M_Sr/(Ca+Sr)_* value increased with the Sr^2+^-substituted content of microarray topography on the HA scaffold surface. In addition, the scaffolds were immersed in cell culture medium, and the cumulative concentration of ions released into the medium is shown in Figure 4. With increasing immersion time, the cumulative ions concentration exhibited an obvious upward trend. The ions concentration also increased with increasing Sr^2+^-substituted content on the HA scaffold surface. Ca^2+^ and PO_4_^3-^ have higher concentration than Sr^2+^. The concentration of Sr^2+^ released in the medium corresponded 8Sr-HA > 4Sr-HA> 1Sr-HA, which is consistent with the Sr^2+^-substituted content in the microarray.

### 3.2. Cell Biocompatibility on the HA Scaffolds with Sr^2+^-Substituted Microarray

**Figure 5:**
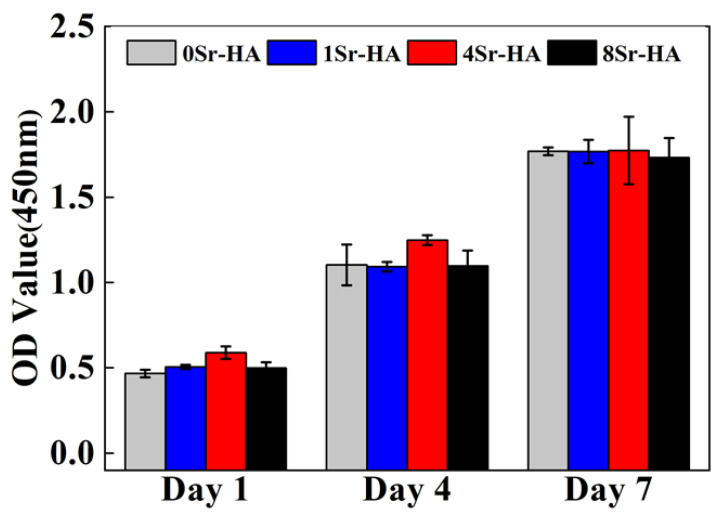
Cell proliferation of ADSCs cultured on HA scaffold surfaces with the Sr^2+^-substituted HA hexagon-like microarray topography for 1, 4, and 7 days.

The CCK-8 assay kit was used to evaluate the proliferation capacity of ADSCs on the scaffold surfaces with Sr^2+^-substituted microarray. As shown in Figure 5, as the culture time extended, the number of ADSCs on the surface of the HA scaffolds continuously increased, and the change in the Sr^2+^-substituted amount on the surface microarray had no obvious negative effect on the proliferation of ADSCs. The results showed that the HA scaffold with Sr^2+^-substituted microarray surface had a good biocompatibility with ADSCs.

**Figure 6:**
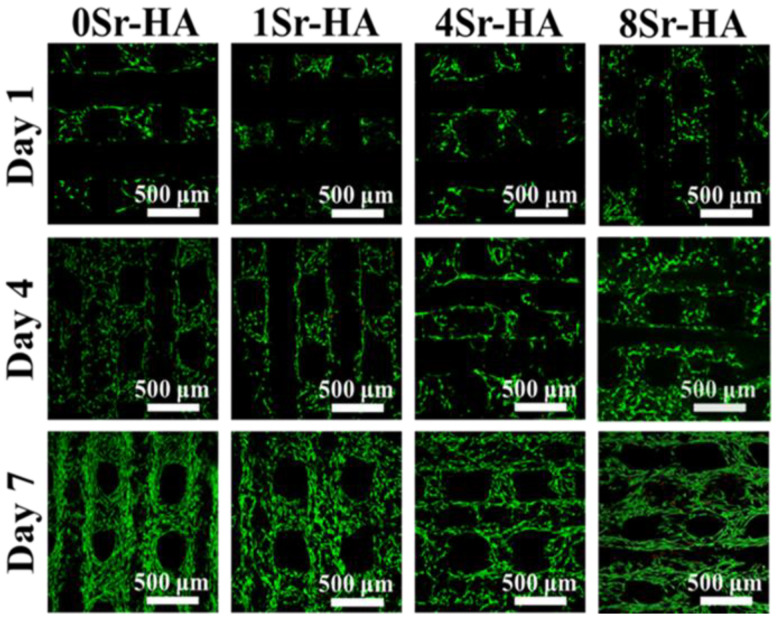
Images of Live/dead staining of ADSCs cultured on the Sr^2+^-substituted HA hexagon-like microarray topography of HA scaffold surfaces for 1, 4, and 7 days.

After ADSCs culture on the HA scaffolds with Sr^2+^-substituted surface microarray for 1, 4, and 7 days, Live/dead staining was carried out to further assess the effect of the Sr^2+^-substituted microarray surface of the HA scaffolds on ADSCs toxicity. The result (Figure 6) showed that ADSCs adhered well on the Sr^2+^-substituted microarray surface of the HA scaffolds, and very few dead cells were found. As the culture time extended to 4 and 7 days, the number of ADSCs on the scaffold surface significantly increased, and the cells evenly covered the scaffold surface evenly and gradually migrated to the pores. Therefore, the Sr^2+^-substituted hexagon-like microarray constructed on the scaffold surface exerted a good bioactive activity toward ADSCs, which is consistent with the results of the proliferation of ADSCs.

**Figure 7:**
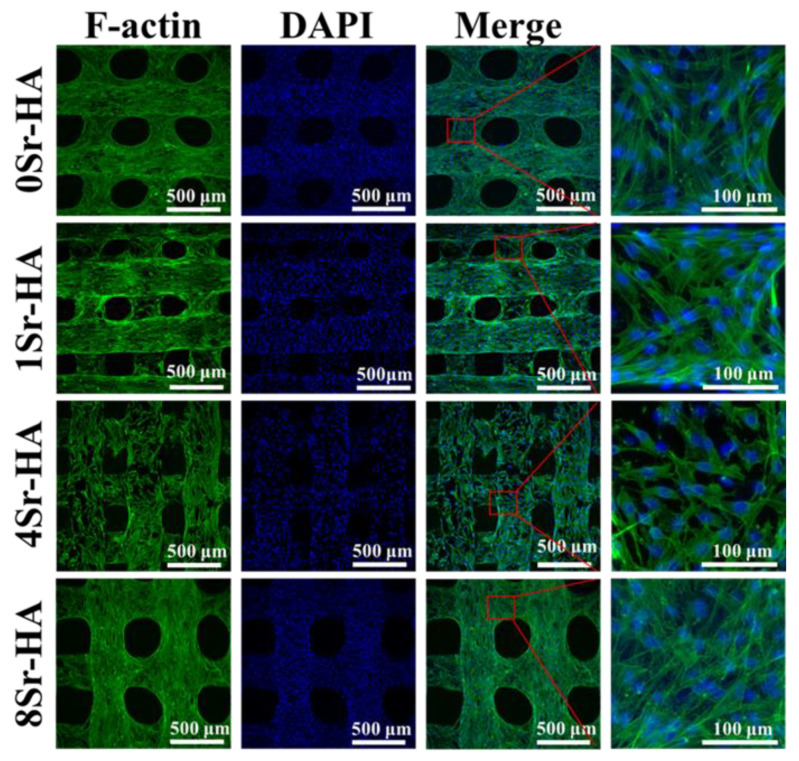
Images of cytoskeletal staining of ADSCs cultured on the Sr^2+^-substituted HA hexagon-like microarray on HA scaffold surfaces for 7 days. The cellular microfilament proteins are stained green. The cellular nuclei are stained blue.

After ADSCs were cultured on the HA scaffolds with Sr^2+^-substituted surface microarray for 7 days, immunofluorescence staining was performed to investigate cell spreading on HA scaffold surfaces with Sr^2+^-substituted HA hexagon-like microarray by the F-actin/DAPI staining (Figure 7). The results showed that the ADSCs proliferated and spread well on HA scaffold surfaces with different Sr^2+^-substituted HA hexagon-like microarray, and spread over the entire scaffold surface after culture for 7 days, demonstrating that the HA scaffold with hexagon-like microarray surface possessed good cytocompatibility and contributed to the spreading of ADSCs.

**Figure 8:**
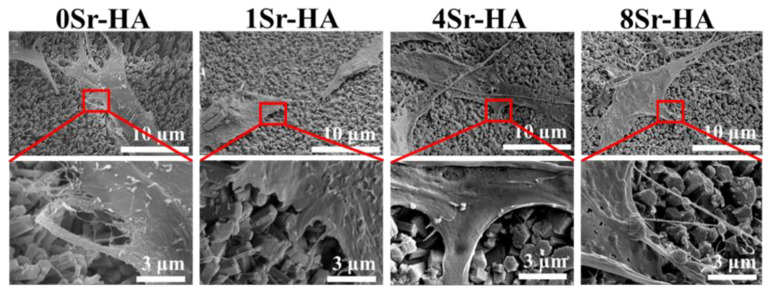
The spreading morphology of ADSCs cultured on the Sr^2+^-substituted HA hexagon-like microarray on HA scaffold surfaces for 1 day.

Subsequently, the morphology of ADSCs attached to the HA scaffolds with the Sr^2+^-substituted surface microarray was evaluated by SEM (Figure 8). ADSCs could spontaneously adhere to the scaffold surface with the Sr^2+^-substituted and filopodia could be clearly observed, indicating that the cells were in a spreading state. These results showed that the HA scaffolds with the Sr^2+^-substituted microarray has good cell compatibility. In addition, white particles were observed on the cell surface, which may be related to the early mineralization process of the cells [32].

### 3.3. Osteogenic Differentiation

**Figure 9:**
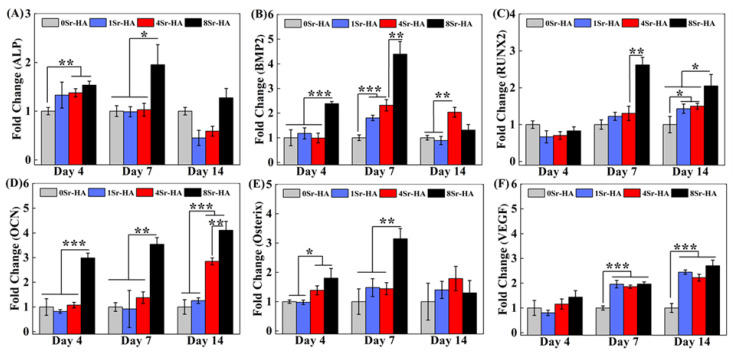
Relative expression levels of marker genes ((**A**), ALP; (**B**), BMP2; (**C**), RUNX2; (**D**), OCN; (**E**), Osterix; (**F**), VEGF) related to osteogenic differentiation of ADSCs cultured on the Sr^2+^-substituted HA hexagon-like microarray on HA scaffold surfaces for 4, 7, 14 days. (* *p* < 0.05, ** *p* < 0.01, *** *p* < 0.001).

It is indispensable for bone regeneration that scaffold accelerate osteogenic differentiation of stem cells. To evaluate the effect of the Sr^2+^-substituted surface microarray on the osteogenic differentiation of ADSCs, the expression levels of marker genes related to osteogenic differentiation were determined by qRT-PCR, as shown in Figure 9.

ALP is an early gene for cellular osteogenesis that can promote the formation of mineralized active bone [36]. After ADSCs were cultured on the scaffold surface for 4 days, the Sr^2+^-substituted HA hexagon-like microarray promoted ALP expression (Figure 9A). After culturing for 7 days, the ALP expression level of ADSCs was higher in 8Sr-HA group than in other groups. The results showed that Sr^2+^-substituted surface microarray can up-regulate the expression level of ALP gene.

Bone morphogenetic protein (BMP) is composed of multifunctional extracellular growth factors in the transforming growth factor-*β* (TGF-*β*) superfamily and is essential for embryonic and adult development [37]. BMP2 is a bone-inducing cytokine that can regulate the adhesion, proliferation, and bone differentiation of bone progenitor cells [35,38]. When ADSCs were cultured on the surface of HA scaffolds for 4 days, BMP2 reached its peak expression level in the 8Sr-HA group (Figure 9B), which was higher than the other groups. Moreover, the expression level of the BMP2 gene also showed an increasing trend with increasing Sr^2+^-substituted at 7 days. These results demonstrated that the Sr^2+^-substituted HA hexagon-like microarray obviously promoted the expression of BMP2. Furthermore, the expression level of BMP2 is closely related to cell osteogenic ability and BMP2 can activate the transcription of RUNX2 and Osterix [39,40].

RUNX2 is a critical controller of osteoblast differentiation and bone formation [35,41], which can regulate the expression of its downstream genes, such as ALP, COLI, OCN and OPN [42,43]. As shown in Figure 9C, at 7 days, the expression level of OCN was obviously up-regulated in the 8Sr-HA group compared with other experimental groups. In the late stage of osteogenic differentiation (14 days), the expression level of RUNX2 was significantly improved in the 8Sr-HA group compared with other experimental groups.

As a bone-specific marker gene during the osteogenic differentiation of cells, OCN gene considered to be the main non-collagen component associated with osteoblast maturation, bone matrix deposition and mineralization, and mainly existed in the osteogenic differentiation anaphase [41,44]. The up-regulated expression of OCN heralds the beginning of extracellular matrix deposition [45]. During the entire cell culture period, the expression level of OCN in ADSCs cultured on the scaffold surface was obviously increased in the 8Sr-HA group compared with the other experimental groups (Figure 9D). At 14 days, the 4Sr-HA group and the 8Sr-HA group exhibited significantly increased the expression level of OCN, which indicated that the Sr^2+^-substituted HA hexagon-like microarray of scaffold surface can endow ADSCs with better bone mineral deposition ability and bone formation ability.

As a significant transcription factor in the early stage of osteoblast differentiation and bone formation, Osterix can regulate the expression of bone-specific matrix protein- associated genes, such as ALP and OCN, and ultimately promote matrix mineralization [40,46]. When cells were cultured on the scaffold surface for 4 days, the expression level of Osterix was significantly higher in the 4Sr-HA group and the 8Sr-HA group than in other groups (Figure 9E); At 7 days, the expression level of Osterix was still significantly increased in the 8Sr-HA group, which may be related to the higher release rate of Sr^2+^ in the 4Sr-HA group and the 8Sr-HA group. Osterix displayed similar expression trends with ALP in the results, which may be because Osterix gene was positively correlated with ALP, and Osterix can regulate the expression of ALP.

As an angiogenesis factor, VEGF not only promotes blood vessel formation, but also osteogenesis during fracture healing and bone defect repair [9,25]. As shown in Figure 9F, in the middle and late stages of cell differentiation, the Sr^2+^-substituted HA hexagon-like microarray significantly promoted the secretion of VEGF.

In summary, the Sr^2+^-substituted HA hexagon-like microarray on the scaffold surface promoted the expression of marker genes related to osteogenic differentiation, which may be due to the higher Sr^2+^ release rate in the 4Sr-HA and 8Sr-HA groups. Moreover, the promotion effect of the scaffold was more obvious in the 8Sr-HA group.

**Figure 10:**
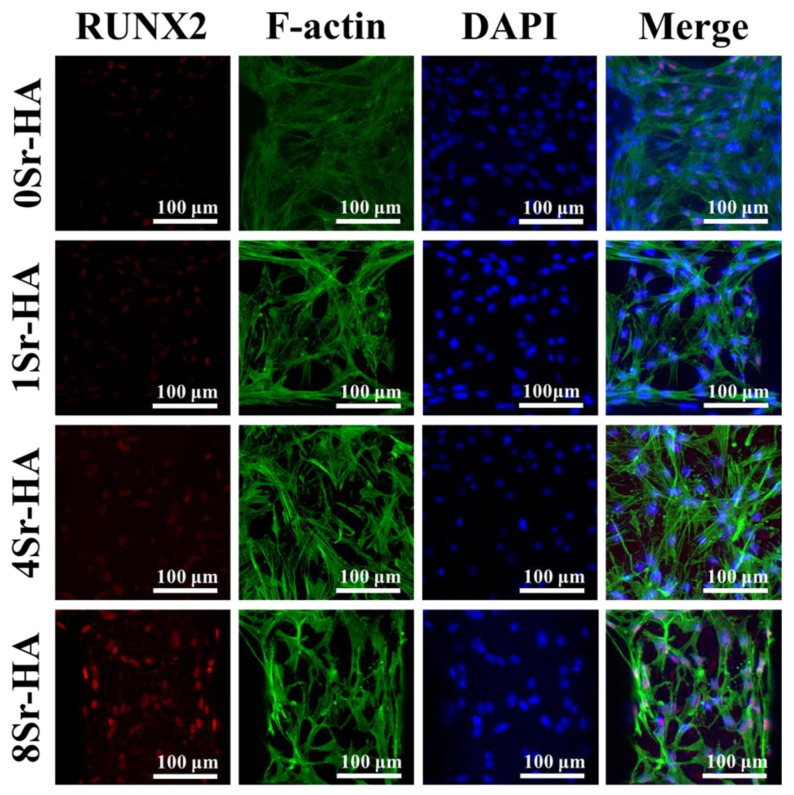
Immunofluorescence staining of a marker protein (RUNX2) of ADSCs cultured on the Sr^2+^-substituted HA hexagon-like microarray topography of HA scaffolds surface for 7 days.

To further observe the effect of the Sr^2+^-substituted HA hexagon-like microarray visually and qualitatively on the osteogenic differentiation of ADSCs, the expression level of marker protein (RUNX2) related to osteogenic differentiation was determined by immunofluorescence staining and observed by CLSM. With the increase of Sr^2+^ content, the expression level of RUNX2 protein showed a visibly increasing trend, indicating that the Sr^2+^-substituted HA hexagon-like microarray can promote stem cells to secrete more RUNX2 protein, which was consistent with the qRT-PCR results. In addition, RUNX2 protein was generally colocalized with the nuclei, which indicated that RUNX2 protein was mainly expressed in the nucleus. (Figure 10). To further evaluate the effect of Sr^2+^-substituted HA hexagon-like microarray on osteogenic differentiation, ADSCs were cultured on the surface of HA porous scaffolds for 7 and 14 days, and the expression levels of marker proteins related to osteogenic differentiation were quantified by Western Blot, the results are shown in Figure S6. When cultured on the surface of the HA scaffold for 7 days, ADSCs could obviously secrete more marker proteins related to osteogenic differentiation (such as ALP, RUNX2, OCN) in the 8Sr-HA group. In the late stage of osteogenic differentiation (14 days), the 8Sr-HA group can still maintain RUNX2 at a high expression level, which demonstrated that Sr^2+^-substituted HA hexagon-like microarray on the HA scaffold surface can promote the secretion of marker proteins related to osteogenic differentiation. The possible mechanism maybe due to the activation of ERK1/2 signaling pathways, which resulted in the modulation of key molecules such as receptor activator of NF-kB Ligand (RANKL) and osteoprotegerin (OPG) that control bone resorption, and to the regulation of genes promoting osteoblastic cell replication, differentiation and survival [35,47].

## 4. Conclusions

We successfully constructed 3D plotted HA scaffolds with interconnected macropore structures. Hexagon-like microarray with different Sr^2+^-substituted amounts was constructed on the 3D-plotted HA scaffold surface through hydrothermal reaction, which was the recrystallization process of Ca^2+^ and PO_4_^3-^. The crystal phase of the Sr^2+^-substituted surface microarray was HA, while the lattice constant of the Sr^2+^-substituted microarray increased with increasing Sr^2+^-substituted content, as the ion radius of Sr^2+^ was higher than that of Ca^2+^. The Sr^2+^-substituted microarray achieved the sustainable release of Sr^2+^. In addition, the Sr^2+^-substituted HA hexagon-like surface microarray had good cytocompatibility and could effectively promote the osteogenic differentiation of ADSCs even without the osteoblast inducing conditional media. In particular, the surface microarray with higher Sr^2+^-substituted (8Sr-HA) had better induction of osteogenic differentiation of ADSCs. HA hexagon-like surface microarray and Sr^2+^-substituted can synergistically improve the osteogenic performance of the HA porous scaffolds. Thus, the Sr^2+^-substituted HA hexagon-like surface microarray on HA scaffolds has the good potential to be used in the construction of bone tissue engineered scaffold.

## Supplementary Materials

The following are available online at https://www.mdpi.com/2079-4991/10/9/1672/s1, Figure S1: The images of the HA scaffold with different shape. (A, B and C), HA scaffolds with different shape; (D), HA scaffolds with different height; (E, F), side and front view of HA scaffold, Figure S2: FE-SEM results of the HA scaffold. (A), fracture surface structure; (B), surface structure of front view and (C) surface topography, Figure S3: XRD (A) and EDS (B) results of the HA scaffold, Figure S4: FE-SEM images about Fracture surface morphology of the HA hexagon-like microarray with different Sr^2+^-substituted contents topographies on the HA scaffold surface, Figure S5: EDS images of the HA hexagon-like microarray with different Sr^2+^-substituted contents topographies on the HA scaffold surface. (the EDS Mapping results were just qualitative and the Sr ions substituted amount in the whole HA scaffold is handful compared with other ions, so the system also would show some lightspots even on the scaffold with no Sr ions substituted), Figure S6: Relative expression of marker proteins related to osteogenic differentiation of ADSCs on the Sr^2+^-substituted HA hexagon-like microarray topography of HA scaffolds surface for (A) 7, (B) 14 days, as measured by western blot, Table S1: The primers for osteogenic related genes, Table S2: The *M_(Ca+Sr)/P_* and *M_Sr/(Ca+Sr)_* value of HA scaffolds with different Sr^2+^-substituted contents by EDS analysis, Table S3: The lattice constant of HA scaffolds with different Sr^2+^-substituted contents.
