# Supplementary material for: Constructing a Sr2+-Substituted Surface Hydroxyapatite Hexagon-Like Microarray on 3D-Plotted Hydroxyapatite Scaffold to Regulate Osteogenic Differentiation
Source: Nanomaterials (Basel). 2020 Aug 26;10(9):1672. doi: 10.3390/nano10091672 (PMC7559340; doi:10.3390/nano10091672)
Supplement: Supplementary file 1 [file nanomaterials-10-01672-s001.pdf]

Supplementary Materials:

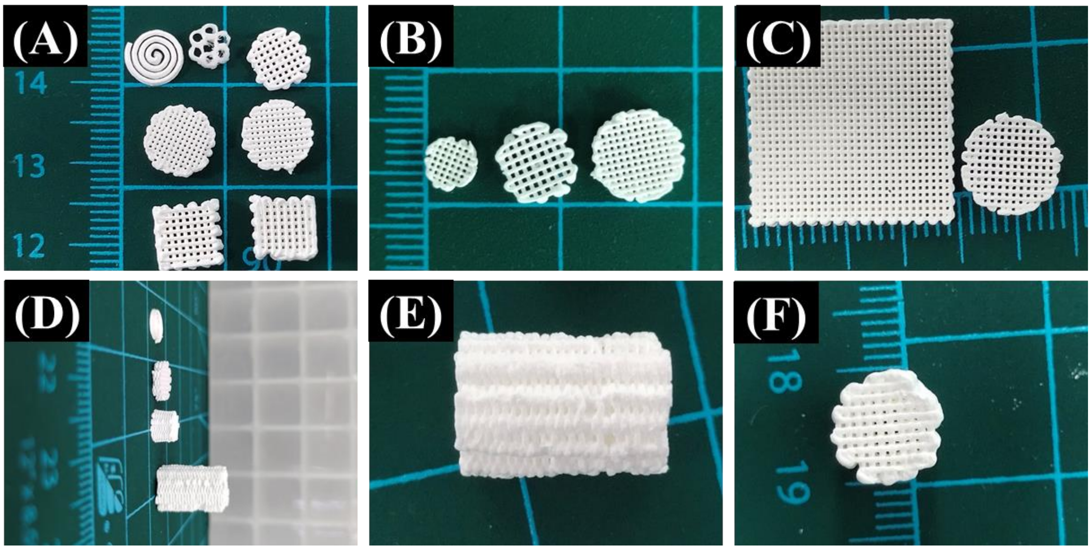

Fig. S1. The images of the HA scaffold with different shape. (A, B and C), HA scaffolds with different shape; (D), HA scaffolds with different height; (E, F), side and front view of HA scaffold.

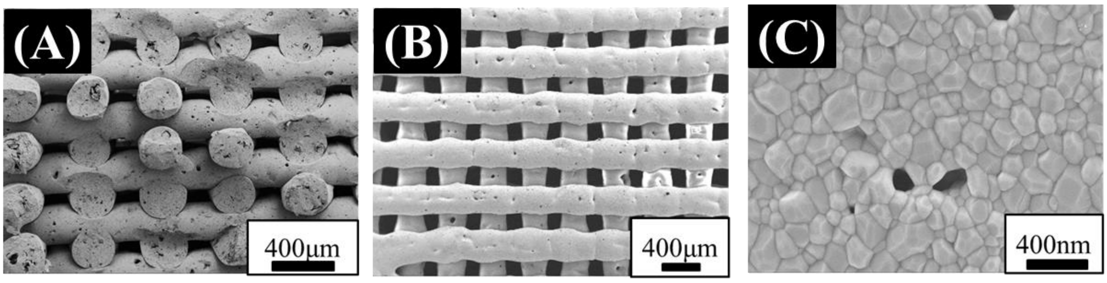

Fig. S2. FE-SEM results of the HA scaffold**.** (A), fracture surface structure; (B), surface structure of front view and (C) surface topography.

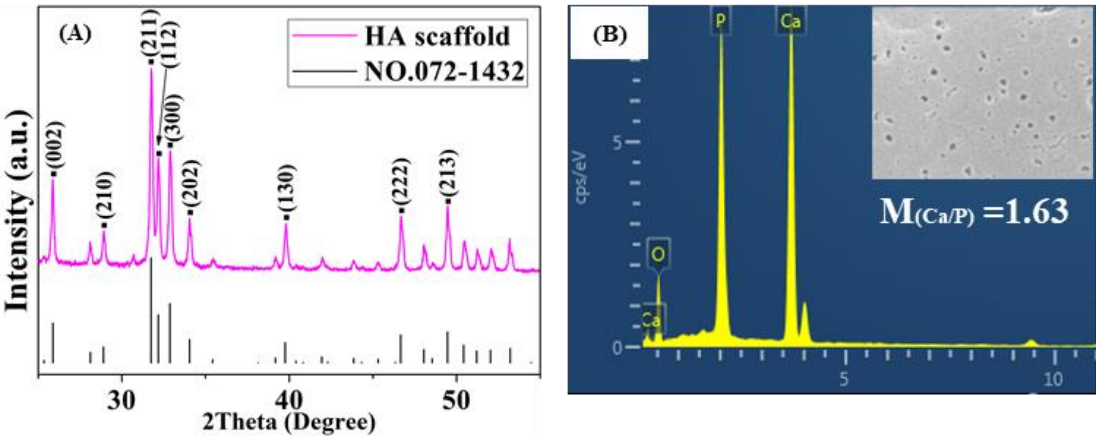

Fig. S3. XRD (A) and EDS (B) re**sults** of the HA scaffold.

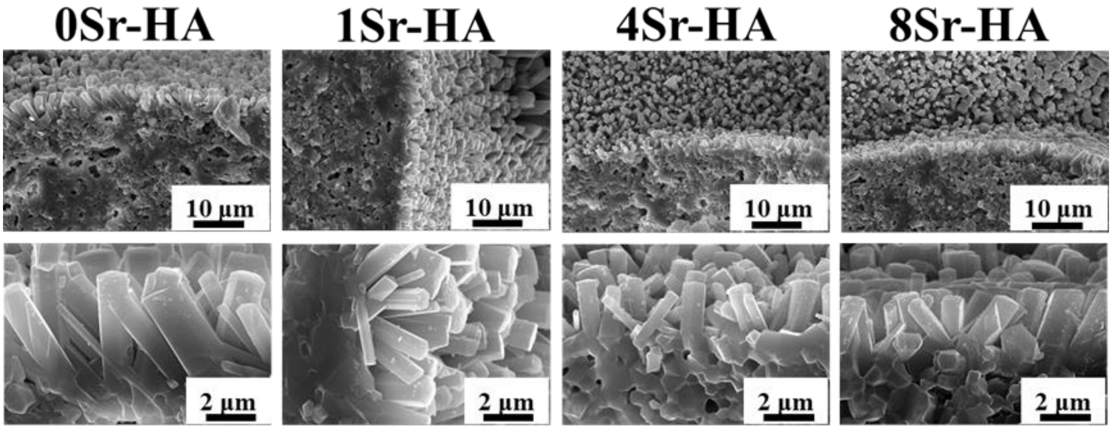

Fig. S4. FE-SEM images about Fracture surface morphology of the HA hexagon-like microarray with different Sr^{2+}-substituted contents topographies on the HA scaffold surface.

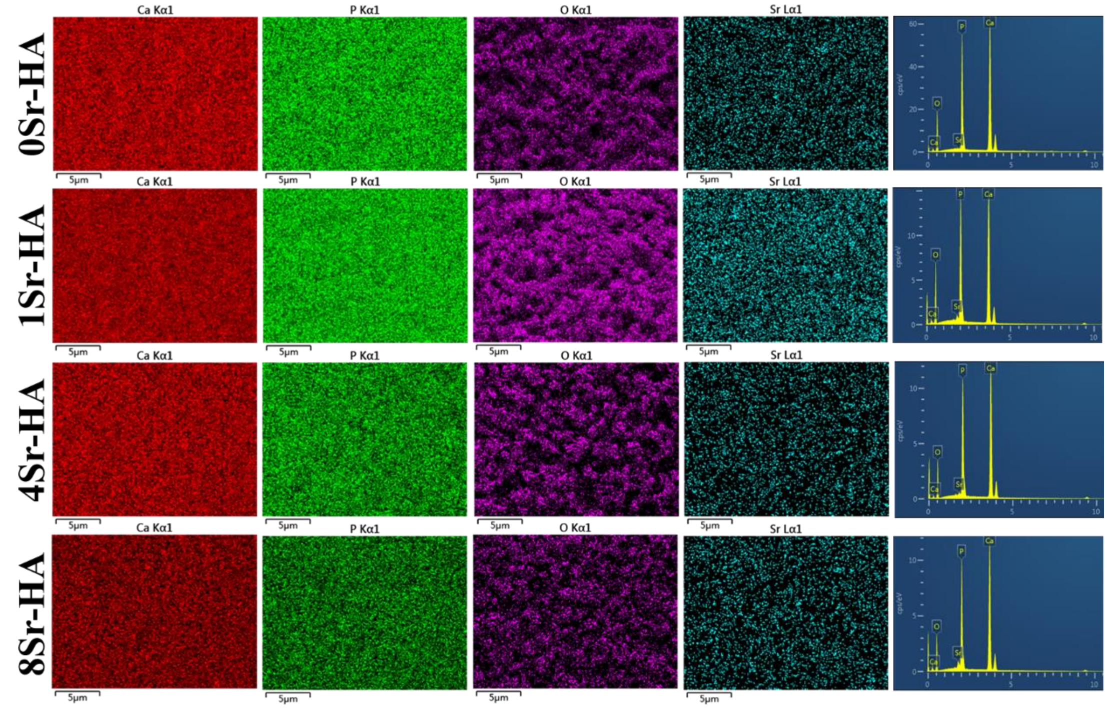

Fig. S5. EDS images of the HA hexagon-like microarray with different Sr^{2+}-substituted contents topographies on the HA scaffold surface. (the EDS Mapping results were just qualitative and the Sr ions substituted amount in the whole HA scaffold is handful compared with other ions, so the system also would show some lightspots even on the scaffold with no Sr ions substituted)

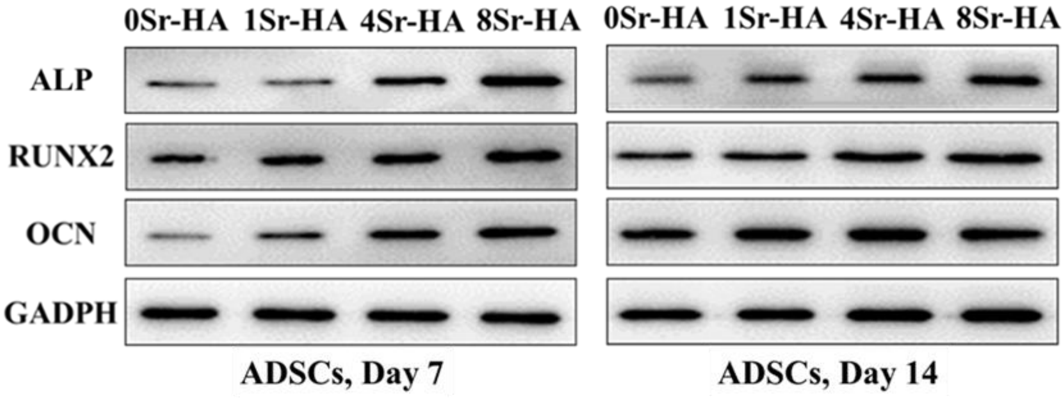

Fig. S6 Relative expression of marker proteins related to osteogenic differentiation of ADSCs on the Sr^{2+}−substituted HA hexagon-like microarray topography of HA scaffolds surface for (A) 7, (B) 14 days, as measured by western blot.

Table S1. The primers for osteogenic related genes.

Target Gene	Direction	Sequence (5′-3′)
GADPH	Forward	TGGATGGCCCCTCCGGGAAA
	Reverse	AGTGGGGACACGGAAGGCCA
ALP	Forward	CACTGGCGGTGCAACAAGA
	Reverse	GAGACCCAATAGGTAGTCCACATTG
BMP2	Forward	AACACTGTGCGCAGCTTCC
	Reverse	CTCCGGGTTGTTTTCCCAC
RUNX2	Forward	CACTGGCGGTGCAACAAGA
	Reverse	CATTCCGGAGCTCAGCAGAATAA
OCN	Forward	CAGCGAGGTAGTGAAGAGA
	Reverse	GACTGGTGTAGCCGAAAG
Osterix	Forward	GCCATTCTGGGCTTGGGTA
	Reverse	TGTGGCAGGGCCAGAGTCTA
VEGF	Forward	TATTCAGCGGACTCACCAGC
	Reverse	CCTCCTCAAACCGTTGGC

Table S2. The $M_{(Ca+Sr)/P}$ and $M_{Sr/(Ca+Sr)}$ value of HA scaffolds with different Sr^{2+}-substituted contents by EDS analysis.

HA scaffold	$M_{(Ca+Sr)/P}$ value	$M_{Sr/(Ca+Sr)}$ value (%)
0Sr-HA	1.62	0.00
1Sr-HA	1.49	0.47
4Sr-HA	1.62	2.97
8Sr-HA	1.72	4.99

Table S3. The lattice constant of HA scaffolds with different Sr^{2+}-substituted contents.

HA scaffold	a（Å）	c（Å）	Volume（nm^3）
0Sr-HA	9.41304	6.87464	1.61913
1Sr-HA	9.41366	6.87515	1.61940
4Sr-HA	9.41687	6.88372	1.62252
8Sr-HA	9.42023	6.88035	1.62289
